# Differences in spinal posture and mobility between adults with obesity and normal weight individuals

**DOI:** 10.1038/s41598-023-40470-5

**Published:** 2023-08-17

**Authors:** Munkh-Erdene Bayartai, Hannu Luomajoki, Gabriella Tringali, Roberta De Micheli, Laura Abbruzzese, Alessandro Sartorio

**Affiliations:** 1 Institute of Physiotherapy, School of Health Professions, Zurich University of Applied Sciences, ZHAW, Katharina-Sulzer-Platz 9, 8401 Winterthur, Switzerland; 2 Department of Physical and Occupational Therapy, School of Nursing, Mongolian National University of Medical Sciences, Ulaanbaatar, Mongolia; 3 Istituto Auxologico Italiano, IRCCS, Experimental Laboratory for Auxo-Endocrinological Research, Piancavallo-Verbania, Italy; 4 Istituto Auxologico Italiano, IRCCS, Division of Eating and Nutrition Disorders, Piancavallo-Verbania, Italy

**Keywords:** Endocrinology, Musculoskeletal system

## Abstract

The aim of this study was to cross-sectionally investigate the relationships between obesity and spinal posture as well as mobility by comparing the spinal parameters between adults with obesity and normal-weight individuals. The spinal parameters were measured in 71 adults with obesity and 142 normal-weight individuals using a radiation-free back scan, the Idiag M360. Differences in spinal posture and movements between the two groups were determined using a two-way analysis of variance. Adults with obesity had greater thoracic kyphosis [difference between groups (Δ) = 6.1°, 95% CI 3.3°–8.9°, *p* < 0.0001] and thoracic lateral flexion (Δ = 14.5°, 95% CI 5.1°–23.8°, *p* = 0.002), as well as smaller thoracic flexion (Δ = 3.5°, 95% CI 0.2°–6.9°, *p* = 0.03), thoracic extension (Δ = 4.1°, 95% CI 1.1°–7.1°, *p* = 0.008), lumbar flexion (Δ = 10.4°, 95% CI 7.7°–13.5°, *p* < 0.0001), lumbar extension (Δ = 4.8°, 95% CI 2.2°–7.4°, *p* = 0.0003) and lumbar lateral flexion (Δ = 12.8°, 95% CI 9.8°–15.7°, *p* = < 0.0001) compared to those with normal weight. These findings provide relevant information about the characteristics of the spine in adults with obesity to be taken into careful consideration in the prescription of adapted physical activities within integrated multidisciplinary pathways of metabolic rehabilitation.

## Introduction

The prevalence of obesity has been increasing across worldwide populations, despite it being preventable^1^. The obesity rate has tripled since 1975. In 2016, more than one-third of adults were overweight, and around one out of 8 adults were obese. The consequences of obesity cover a wide range of health issues and the associated economic burden in both developed and developing countries^1–3^. A systematic review of the global economic burden of obesity reported that obesity-related costs across different countries range between 0.7% and 2.8% of their health expenditures, whilst medical costs were around 30% greater in people with obesity than in those who were not obese^4^. Systematic reviews of health and economic consequences of obesity also highlight that an urgent need for improved strategies for better prevention and intervention of obesity as a chronic disease^2,3^. Various body systems are negatively affected by obesity, leading to an increased risk of pathological conditions, including metabolic, cardiorespiratory, and musculoskeletal diseases^1,3^. For example, obesity is considered to be one of the five metabolic factors, including high blood pressure, hyperglycemia, central obesity, high triglycerides, and abnormal cholesterol that raise the risk of cardiovascular disease and type 2 diabetes^5^. The increasing prevalence of obesity worldwide is considered by International Diabetes Federation as one of the main driving factors behind the high prevalence of the metabolic syndrome^6^. Physical activity, diet and behavioural changes remain the cornerstone of the management of obesity and obesity related conditions^7,8^ as overweight and obesity are more likely if there is an imbalance between energy intake and expenditure^9^. Promoting exercise and motion commonly used in the management of obesity is also associated with a reduced risk of common musculoskeletal conditions, including low back pain and knee osteoarthritis^8,10,11^. However, the influence of obesity on human motion, particularly spinal kinematics/movements, although crucial to daily activities and the management of spinal conditions, has been understudied.

Spinal kinematics/motion appears to be altered by obesity in both adolescents and adults^12,13^. Spinal kinematics/motion is essential to perform a range of activities of daily living, such as personal hygiene activities (hand washing, washing hair and shaving) and locomotive activities of daily living (walking, ascending and descending stairs)^14^. Putting on socks and picking up an object off the floor were found to be the activities of daily living requiring the largest lumbar range of motion compared to other activities of daily living^14^. Alterations of spinal movements are also often linked with musculoskeletal problems, particularly low back pain. To date, both cross sectional and longitudinal studies found that alterations in spinal kinematics are associated with low back pain^15–17^. Abnormal loading in the spine leading to tissue degeneration, potentially causing pain and other symptoms may be caused by alterations in spinal kinematics^18^. In clinical practice, spinal posture and flexibility/motion are also commonly investigated as part of musculoskeletal examinations, emphasising the importance of preserving spinal kinematics. Additionally, obesity is believed to be a risk factor for developing low back pain, although the mechanism through which obesity influences low back pain remains unclear. A cross-sectional study of nine healthy normal-weight adults and nine obese individuals investigating differences in spinal segment range of motion found that obese adults had smaller lumbopelvic motion compared to age, height and sex matched normal weight individuals^13^. However, studies examining the influence of obesity on spinal kinematics/motion, particularly spinal postures remain sparse to date. Exploring the effect of obesity on spinal postures as well as kinematics/motion would help to better understand the characteristics of these spinal parameters in obese adults.

The aim of this study was to examine the influence of obesity on spinal postures as well as kinematics/motion and hip kinematics by comparing the parameters in obese adults and in normal weight individuals.

## Methods

The present study used a cross-sectional design to examine the relationship between obesity and spinal posture as well as mobility and followed the “Strengthening the Reporting of Observational Studies in Epidemiology (STROBE)” recommendations^19^.

### Participants

Obese and normal-weight individuals according to the World Health Organization guidelines were recruited into the study^20^. Obesity and normal weight were defined in presence of a body mass index > 35 and BMI < 25, respectively. Participants were excluded if they had past and present musculoskeletal or neurological conditions, such as limb length discrepancy, spinal deformities and surgeries determining physical disabilities, as well as those taking anti-inflammatory medications. Normal-weight individuals were recruited from the general population in the Canton of Zurich, Switzerland. Individuals with obesity who were hospitalized for a three-week multidisciplinary body weight reduction program at the Division of Metabolic Diseases, Istituto Auxologico Italiano, IRCCS, Piancavallo (VB), Italy were recruited in this study. The Ethics Committee of the Istituto Auxologico Italiano (Milan, Italy; research project code: 01C124; acronym: PRORIPONATFIS) and the Ethics Committee of Zurich (BASEC-no. 2018-00979) approved the study. All procedure in this study were in compliance with the Helsinki Declaration of 1975, as revised in 2008. The purpose of the study was explained to the participants and written informed consent was obtained.

### Measurements

Posture and mobility of the spine and hip motion were assessed using the Idiag M360 scan tool (Idiag, Fehraltorf, Switzerland)^21^. Spinal postures were defined from an upright standing position, whilst spinal mobility/range of motion was determined during dynamic tasks, such as flexion, extension and lateral bending. The Idiag M360 is a radiation-free, non-invasive, reliable, skin-surface device, which quantifies the spinal posture and motion through recording angles of each vertebral joint and sacral slope using computer-assisted analysis. The data of these parameters are sampled at a frequency of 150 Hz^22,23^. During the recording, positions and mobility of each individual segment are estimated. Vertebral distances and angles are measured while two rolling wheels contained in the device follow the vertebral spinous processes. An analogue–digital converter transfers radiographically the parameters, estimated by the rolling wheels, to a personal computer. A validity study evaluating for measurements of spinal curvatures using an X-ray examination showed a very good correlation between measurements made by radiography and spinal mouse^22,24^. Spinal ranges of motion reported in the literature previously were also comparable with the values determined by the Idiag M360^25^. Additionally, a previous comparative study exploring the validity and reliability of the device for the measurement of lumbar flexion also reported that segmental and global lumbar ranges of motion determined by radiography and the device were comparable^26^.

The reliability of the device has previously been examined in both normal weight and obese individuals^12,27^.

The spinal parameters (spinal and hip mobility) were determined in the longitudinal and coronal planes as participants were instructed to do flexion (bend forward), extension and then bilateral side bending from an upright standing position, as described in the protocol for measuring spinal posture and mobility provided by previous studies^12,22,27^. Using the educational videos, both assessors who evaluated the spinal posture and mobility in the obese and normal weight participants had been trained by the Idiag staff.

The reliability of the device in obese individuals was examined as part of the previous study, showing that the intrarater intraclass correlation coefficients for the spinal postures ranged from 0.86 to 0.94, and the standard error of measurement values ranged 0.58° to 0.70°^12^, whilst for spinal mobility the intrarater intraclass correlation coefficients in the coronal and sagittal planes ranged from 0.57 to 0.80 and 0.87 to 0.98, respectively. Measurements of spinal curvature and kinematics/motion using the device in non-obese individuals also showed fair to good reliability^25^. In this study, the average standard error of measurement was approximately 2°, whilst the interexaminer reliability on lumbar and thoracic movements assessed by two examiners ranged from 0.62 to 0.93^25^.

### Data processing

The difference between range of motion values in each segment of the spine determined at the standing position and the end of motion ranges in the sagittal and frontal planes was used to estimate each range of motion value for the spinal parameters. The sum of the respective range of motion values in each spinal segment (5 and 12 range of motion values for the lumbar and thoracic spine, respectively) were used to determine the total lumbar and thoracic range of motion of the spine. The sacral inclination at the end of lumbar flexion and extension in the sagittal plane was used to determine hip flexion and extension, respectively.

### Statistical analysis

R version 4.2.2 was used to perform descriptive and inferential statistics^28^. Mean values and standard deviations (SD) for participants’ age, sex, spinal posture and motion, as well as hip motion and lumbar to hip ratio were determined in descriptive statistics. Shapiro–Wilk test was used to assess data normality. Differences in demographic and anthropometric characteristics between the two groups (obese and normal-weight) were determined using the independent samples t-test for normally distributed data, the Wilcoxon’s rank sum test for non-normally distributed parameters and the Pearson’s chi-square test for categorical variables. Multiple regression models adjusting for age and sex were used to explore the association between BMI and spinal movements. Age and sex adjusted two-way analysis of variance (ANOVA) was used to determine statistically significant differences in spinal posture and mobility between the two groups. Pairwise post hoc tests were applied using the software package “emmeans” to compare between groups following the ANOVA tests^29^. Statistical significance was considered as a p value of less than 0.05.

## Results

**Figure 1:**
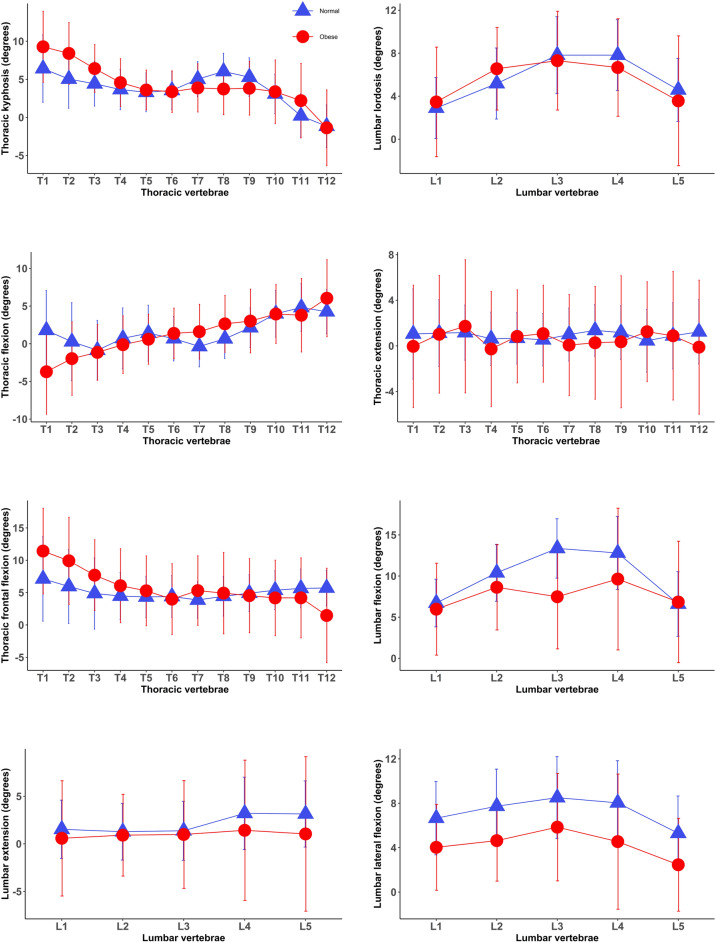
Posture and motion/kinematics of each individual spinal segment in the normal weight and obese groups (mean and standard deviations). Positive and negative values indicate kyphosis/flexion and lordosis/extension, respectively. Normal—normal-weight group, Obese—obese group.

A total of 71 adults with obesity (45 females, 26 males) and 142 normal-weight individuals (91 females, 51 males) participated in the study. Participants’ characteristics are shown in Table 1, and no differences in the mean age, height and sex ratio were not observed between the normal-weight and obese groups (*p* > 0.05). Segmental posture and motion of the spine in the normal-weight subjects in comparison with individuals with obesity are described in Fig. 1. Differences in segmental postures and movements of proximal thoracic vertabrae were most commonly observed between the two groups. For instance, larger kyphosis and less flexion in proximal thoracic vertabrae were observed in adults with obesity than in normal-weight individuals (Fig. 1).

**Table 1:** Participants’ characteristics in the normal-weight and obese groups (mean ± standard deviations).

Variables	Normal-weight adults (N = 142)	Adults with obesity (N = 71)	*p* value
Age (years)	45.3 (13.8)	47.8 (15.2)	0.16^w^
Sex (female)	64%	63%	0.53^c^
Weight (kg)	60.2 (7.3)	121.5 (24.5)	< 0.0001^w^
Height (cm)	168.9 (8.1)	166.3 (9.2)	0.06^w^
BMI (kg/m^2^)	21.1 (1.1)	43.6 (6.6)	< 0.0001^w^

*p* value—the significance of differences between the two groups was calculated by ^(w)^Wilcoxon’s rank sum test and the ^(c)^Pearson’s chi-square test.

*BMI* body mass index.

In the age and sex adjusted regression analysis on BMI and spinal movements of adults with obesity and normal-weight individuals, thoracic flexion (β = − 0.24, 95% confidence interval [CI] − 0.37; − 0.10), lumbar flexion (β = − 0.37, 95% CI − 0.50; − 0.24), lumbar extension (β = − 0.23, 95% CI − 0.34; − 0.13), lumbar lateral flexion (β = − 0.54, 95% CI − 0.66; − 0.42), hip flexion (β = − 0.48, 95% CI − 0.67; − 0.29) and hip extension (β = − 0.28, 95% CI − 0.42; − 0.14) were inversely associated with body mass index except for thoracic lateral flexion (β = 0.76, 95% CI 0.38; 1.14), which was positively associated with body mass index. No statistically significant association was found between thoracic extension and BMI (*p* > 0.05).

### Associations of obesity with spinal posture and mobility as well as hip motion

Statistically significant differences were observed in thoracic kyphosis and spinal as well as hip mobility between the normal-weight and obese groups (Table 2). Individuals with obesity had larger thoracic kyphosis, particularly proximal thoracic kyphosis than the normal-weight subjects, whilst distal thoracic kyphosis was smaller in the obese participants. No statistically significant differences were observed in lumbar lordosis and sacral kyphosis between the two groups. Spinal and hip mobility were smaller in the obese participants than the normal-weight subjects except for the thoracic lateral flexion, which was greater in the individuals with obesity. In the post hot test, the greatest differences observed among spinal mobility were thoracic and lumbar lateral flexion (Table 2). Thoracic lateral flexion was 14.3° greater in obese individuals, whereas lumbar lateral flexion was 13.80 larger in normal-weight subjects. No statistically significant differences were observed between the two groups in lumbar lordosis, sacral kyphosis and lumbar to hip ratio.

**Table 2:** Differences in spinal postures as well as motion/kinematics and hip mobility between the normal-weight and obese groups.

Variables	Normal weight adults (N = 142)		Adults with obesity (N = 71)		Differences in spinal posture and kinematics (95% CI)	*p* value
	EMM	SE	EMM	SE		
Spinal postures						
Thoracic (°)						
Kyphosis (Th1-12)	45.6	0.8	51.7	1.1	− 6.1 (− 8.9 to − 3.3)	< 0.0001
Proximal thoracic kyphosis (Th1-6)	26.2	0.6	35.3	0.9	− 9.1 (− 11.3 to − 6.9)	< 0.0001
Distal thoracic kyphosis (Th7-12)	19.2	0.7	15.7	1.0	3.5 (0.9 to 6.0)	0.007
Lumbar lordosis (°)	27.4	0.7	27.7	1.0	− 0.3 (− 2.8 to 2.2)	0.82
Sacral kyphosis (°)	13.9	0.6	15.0	0.9	− 1.1 (− 3.3 to 1.2)	0.34
Spinal mobility						
Thoracic (°)						
Flexion	19.1	1.0	15.6	1.4	3.5 (0.2 to 6.9)	0.03
Extension	11.7	0.9	7.6	1.2	4.1 (1.1 to 7.1)	0.008
Lateral flexion	62.1	2.7	76.6	3.9	− 14.5 (− 23.8 to − 5.1)	0.002
Lumbar (°)						
Flexion	49.5	0.9	39.1	1.3	10.4 (7.7 to 13.5)	< 0.0001
Extension	10.4	0.7	5.6	1.0	4.8 (2.2 to 7.4)	0.0003
Lateral flexion	35.6	0.8	22.8	1.2	12.8 (9.8 to 15.7)	< 0.0001
Hip mobility						
Hip (°)						
Flexion	58.0	1.3	46.2	1.9	11.8 (7.3 to 16.4)	< 0.0001
Extension	13.8	1.0	6.9	1.4	6.9 (3.5 to 10.3)	0.0001
Lumbar to hip ratio	0.47	0.01	0.45	0.01	0.02 (− 0.01 to 0.05)	0.34

*p* value (adjusted for age and sex)—the significance of differences between the two groups. EMM estimated marginal means for the sum of the respective range of motion values in each spinal segment in the different spinal regions. SE standard errors. CI confidence interval. Lumbar to hip ratio was calculated by dividing lumbar range of motion by the sum of lumbar range of motion and hip range of motion during the trunk flexion in the sagittal plane.

## Discussion

The purpose of the present study was to investigate the relationship between obesity and spinal posture as well as motion/kinematics in adults, with the key finding being that alterations in spinal posture and mobility as well as hip motion were associated with obesity. Obesity in adults was associated with decreased spinal and hip mobility and increased thoracic kyphosis, particularly proximal kyphosis but decreased distal thoracic kyphosis and lateral flexion. Thoracic kyphosis appears to be substantially influenced by obesity, which may in turn increase mechanical challenges that the thoracic spine must withstand.

Increased thoracic kyphosis was associated with obesity, but no significant associations were found between obesity and lumbar lordosis as well as sacral kyphosis. Although the excess of fat mass is generally believed to add strain to the spine, studies specifically examining the association of obesity with spinal postures in adults are lacking but alterations in spinal postures have been explored in relation to obesity in children and adolescents^12,30^. For instance, a cohort study of 1621 adolescents with and without idiopathic scoliosis examining retrospectively the association of BMI with thoracic kyphosis showed that the relationship between increased BMI and increased thoracic kyphosis in both adolescents with and without spinal deformity^30^. Our previous study examining the influence of obesity on spinal posture in children and adolescents also found the association of obesity with increased thoracic kyphosis^12^, suggesting that obesity may play an important role in the characteristics of spinal postures in both children and adults, regardless of age. Additionally, these findings also imply that the influence of obesity determined in children and adolescents on thoracic kyphosis may be preserved in adulthood. Obesity was not associated with lumbar and sacral postures in the current study, which was consistent with the findings from previous cross-sectional studies exploring the impact of BMI and obesity on spino-pelvic parameters, including lumbar lordosis and sacral slope, measured on lateral X-ray^31,32^. A previous cross-sectional study of female participants (30 normal-weight, 30 overweight and 29 obese) examining the impact of age, BMI and the bone mineral density on lumbar and pelvic postures demonstrated no differences in the vertebral and spinopelvic angle measures between the three different BMI groups^31^. Another cross-sectional study of individuals who were assigned into the three different BMI groups (51 normal weight individuals, 93 overweight, and 56 obese) exploring the association between obesity and the spino-pelvic characteristics revealed that no significant differences were observed in lumbar lordosis as well as sacral slope across the three different BMI groups^32^. These results suggest that obesity appears to affect thoracic kyphosis more than lumbar and sacral postures, regardless of age. Both cross-sectional and longitudinal studies have shown that hyperkyphotic posture is associated with reduced physical function, such as reduced gait speed as well as impaired functional mobility and physical performance^33–37^. These results imply that the combination of obesity and increased risk of hyperkyphotic posture could potentially further worsen physical performance and mobility. However, future longitudinal studies are needed to deepen the understanding of potential impacts of obesity on physical performance and mobility. In addition, alterations in thoracic kyphosis appears to increase with aging as a systematic review examining the posture and motion of the thoracic spine reported that thoracic kyphosis deepens by approximately 3 degrees per decade^38^. In the present study, thoracic alignment of participants in both the obese and normal-weight groups also was hyperkyphotic as the mean angle of the thoracic kyphosis was more than 40°^39^. A change in thoracic kyphosis is also observed in the transition from sitting to standing position^40^^,^^41^. For instance, a prospective study reported that a decrease of 8.5° in thoracic kyphosis in the transition between sitting and standing positions due to the change in the sagittal balance and the center of gravity from standing to sitting^40^. A recent review study also summarized that the thoracic kyphosis, lumbar lordosis and sacral slope decreased from standing to sitting by up to approximately 50% as sitting leads to straighten the spine^41^. These findings would help to better understand spinal posture and kinematics/motion in relation to obesity and provide further insights into mechanical challenges associated with obesity such as excessive accumulation of body fat in the trunk, increasing the mechanical load of the thoracic spine. However, future studies exploring the effect of obesity on alterations in spinal posture longitudinally could be important to better understand the role of obesity in spinal posture and its deformity.

The spinal and hip movements measured in the sagittal and frontal planes were smaller in the obese adults than in the normal-weight individuals, except for thoracic lateral flexion, which was larger in the obese participants. Spinal and hip movements were also inversely associated with BMI, except for thoracic lateral flexion, which was positively associated with BMI. Reduced spinal mobility in adults with obesity could mainly be explained by their dimensions potentially restricting the available range of motion at the hip and the spine. For example, a previous retrospective of 84 patients stratified into normal, overweight and obese groups found that individuals with obesity demonstrated more posterior spinopelvic tilt from standing to sitting to compensate for soft-tissue tension around the hip joint limiting hip flexion^42^. However, more research is needed to better understand whether there are other mechanisms/factors associated with obesity contributing to restrict spinal mobility other than the body dimensions or physical features of obesity. Spinal mobility is crucial for performing daily activities and reduced spinal movements are often linked with musculoskeletal problem, particularly low back pain, suggesting the importance of preserving spinal mobility^14,17,43^. For example, a systematic review of prospective studies exploring the association of reduced spinal mobility with low back pain demonstrated that restricted lumbar mobility in the coronal plane was associated with the risk of low back pain^17^. Previous studies exploring differences in the characteristics of the spine between obese and normal-weight people also revealed that individuals with obesity had smaller spinal mobility^30^^,^^44^. Additionally, a systematic review of in vivo studies investigating thoracic posture and motion in adults with and without idiopathic scoliosis highlighted decreased thoracic range of motion in obese individuals^30^, which was consistent with the findings from the present study. However, in the present study, thoracic lateral flexion was greater in adults with obesity whereas lumbar lateral flexion was smaller in these participants compared to the normal-weight adults. Obesity appears to have an influence on decreased lumbar lateral flexion, which in turn may require increased thoracic lateral flexion. Our previous study conducted in obese and normal-weight children and adolescents showed that both thoracic and lumbar lateral flexion movements were smaller in the obese participants^12^, suggesting that smaller spinal lateral movements needed for daily activities may be compensated by increased thoracic lateral flexion. Nevertheless, future longitudinal studies following adolescents until their adult age may be important to better understand how obesity impacts on the characteristics of the spine from childhood to adulthood.

Obesity was associated with reduced hip mobility as well, but no associations were found with lumbar to hip ratio. Hip mobility is also important for our daily activities that allows individuals to move around in the environment. Hip range of motion is crucially important to conduct activities of daily living and functional activities requiring a large range of motion such as squatting, kneeling, as well as cross-legged sitting^43^. For example, a previous cross-sectional study of generating three-dimensional kinematics at the hip, knee and ankle joints of 30 healthy participants when performing high range of motion activities, namely (squatting heels down, squatting heels up, kneeling dorsi-flexed, kneeling plantar-flexed and sitting cross-legged) reported that squatting with heels up required the largest hip flexion, reaching up to 95 degrees, whereas kneeling plantar-flexed required the smallest hip flexion, reaching up to 62 degrees among the five activities of daily living^43^. Therefore, obesity could potentially hinder adults with obesity more in performing functional activities, such as squatting compared to those with normal weight.

Restrictions in hip mobility are also associated with musculoskeletal conditions, particularly hip conditions. For example, a systematic review of prospective and case control studies determined the association of reduced total hip range of motion, particularly hip rotation with the risk of developing groin pain in athletes^45^. A previous study of 30 female participants with non-specific low back pain exploring the effect of stretching exercises targeting the hip flexors for a period of 8 weeks revealed that disability, pain, and passive range of motion improved more in participants who received the exercises than those who did not this intervention^46^. The findings of this study suggest that hip flexibility, particularly hip extension may be crucial to be taken into account in the management of non-specific low back pain. In addition to reduced hip flexibility in people with obesity, the combination of hyperkyphotic posture and reduced spinal mobility could potentially further contribute to increase the risk of low back pain. Although hip mobility was smaller in adults with obesity, lumbar to hip ratio was not different between adults with obesity and normal weight controls. Decreased range of motions observed in both the hip and lumbar mobility could explain this as the proportion of these two variables defines the lumbar to hip ratio. Our previous study conducted in children and adolescents also demonstrated that obesity was associated with reduced hip mobility but not with lumbar to hip ratio, implying that the influence of obesity on hip mobility may also be preserved in adulthood^12^. However, a need for future studies to investigate whether longitudinal associations between obesity and hip mobility exist to further understand the role of obesity in hip mobility.

We acknowledge that the present study has several limitations. The present study employed the cross-sectional design, which precludes any causal interpretations of the observed relationships between obesity and spinal posture as well as motion/kinematics. In the present study, spinal mobility was measured in the sagittal and frontal planes, thus hampering to generalise the influence of obesity on the spinal movements measured to spinal mobility in the transverse plane. The accuracy of the Idiag M360 as a skin surface device for the measurement of spinal posture and mobility could potentially be affected by skin movement artefact, particularly in the obese participants due to a greater percentage of soft tissue. However, the study was strengthened by providing detailed information about the characteristics of the spine such as global as well as segmental posture and motion of the spine in adults with obesity and normal weight controls. In addition, the relatively broad study population (obese and controls) was evaluated in the two research centers by well-trained staff in standardized experimental conditions.

Obesity was associated with changes in spinal posture, and spinal as well as hip motion/kinematics in adults. Obesity was associated with increased thoracic kyphosis but decreased spinal and hip mobility, except for thoracic lateral flexion. Increased thoracic kyphosis observed in those with obesity may be a biomechanical adaptation to maintain center of gravity in the anterior–posterior direction. Reduced lumbar lateral movements found in the adults with obesity may be compensated by increased thoracic lateral flexion to perform for daily activities. Lumbar lordosis and sacral kyphosis or the lumbar to hip ratio appear to be not affected by obesity. These results provide relevant important information about the characteristics of the spine in adults with obesity to be taken into careful consideration in the prescription of adapted physical activities within integrated multidisciplinary pathways of metabolic rehabilitation, as well as unique insights into mechanical challenges associated with obesity such as excessive accumulation of body fat in the trunk, increasing the mechanical load of the spine. These findings also highlight the significance of considering obesity in the musculoskeletal assessment of spinal curvatures and approaches designed to promote spinal and hip flexibility in adults with obesity.

## Data Availability

The data presented in this study are available from the corresponding author upon reasonable request.
